# Physico-Mechanical Properties of Commercially Available Tissue Conditioner Modified with Synthesized Chitosan Oligosaccharide

**DOI:** 10.3390/polym14061233

**Published:** 2022-03-18

**Authors:** Asfia Saeed, Shahreen Zahid, Muhammad Sajid, Shahab Ud Din, Mohammad Khursheed Alam, Farooq Ahmad Chaudhary, Muhammad Kaleem, Haytham Jamil Alswairki, Huda Abutayyem

**Affiliations:** 1 Department of Dental Materials, Army Medical College, National University of Medical Sciences, Rawalpindi 46000, Pakistan; asfiasaeed@hotmail.com (A.S.); dr_kaleem78@hotmail.com (M.K.); 2 Department of Dental Materials, Islamabad Medical & Dental College, Islamabad 45400, Pakistan; m.sajid@iideas.edu.pk; 3 Department of Dental Materials, Dental College HITEC-IMS, Taxilla 751010, Pakistan; shahreen.khan@gmail.com; 4 School of Dentistry (SOD), Federal Medical Teaching Institution (FMTI)/PIMS, Shaheed Zulfiqar Ali Bhutto, Medical University (SZABMU), Islamabad 44000, Pakistan; drshahab728@hotmail.com; 5 Preventive Dentistry Department, College of Dentistry, Jouf University, Sakaka 72345, Saudi Arabia; 6 School of Dental Sciences, Universiti Sains Malaysia, Kota Bharu 16150, Malaysia; hitham.swerki@gmail.com; 7 Department of Orthodontics, College of Dentistry, Ajman University, Ajman 346, United Arab Emirates; h.abutayyem@ajman.ac.ae

**Keywords:** antifungal chitosan, tissue conditioning, sorption, Shore A hardness, denture stomatitis

## Abstract

This study aims to compare the hardness, sorption and solubility of commercially available tissue conditioner [TC] modified with chitosan [CS] and synthesized chitosan oligosaccharide [COS] in antifungal concentration. COS was synthesized by acid hydrolysis and characterized by FTIR and XRD. Experimental materials were formulated by incorporating each per gram of TC powder with effective antifungal concentration of chitosan 1.02 mg (Group 1: TC-CS) and 0.51 mg COS (Group 2: TC-COS). A commercially available TC was used as control (Group 0: CTC). Shore A hardness test was performed according to ASTM D 2240-05 (2010) standards on samples stored in dry environment, distilled water (DW) and artificial saliva (AS) at 37 °C (*n* = 5 per group). Percent weight changes (*n* = 5 per group) after storage in DW and AS was used to record sorption and solubility. One-way Anova with post hoc Tukey’s test was applied. FTIR and XRD confirmed low molecular weight and amorphous nature of COS. Experimental groups had higher Shore A hardness values; however, these changes were not significant. Greatest variations in durometer values (*p* ≤ 0.05) were observed during the first 24 h. Experimental groups had higher (*p* ≤ 0.05) percentage sorption and solubility. Samples stored in DW had significantly higher (*p* = 0.019) sorption, whereas material had higher (*p* = 0.005) solubility in AS. Mean solubility values in both immersion mediums was highest for Group 2, followed by group 1 and group 0. In addition, significant (*p* ≤ 0.05) increase in solubility upon aging was noted for each material. Experimental tissue conditioner had higher hardness, sorption and solubility. However, these changes are not substantial to interfere with their tissue healing property. Therefore, these materials may be considered and explored further as potential antimicrobial drug delivery agent for denture stomatitis patients.

## 1. Introduction

Denture stomatitis is a pathological condition affecting approximately 65% of denture wearers [1,2]. Among these patients, 93% suffers from candida infections [1]. Prescription of topical antifungal medicaments has been recommended to treat such infections [3]. However, in geriatric patients, the effectiveness of treatment is compromised by their limited motor skills, short term memory and special needs [4,5]. To address this issue, use of tissue conditioners as a drug delivery vehicle has been explored [4]. Tissue conditioners allow direct, precise, and sustained availability of an antifungal medicament at the affected site without requiring patient compliance. In addition, its resilient nature helps to absorb mechanical stresses generated during mastication and allow healing of traumatized tissues [4].

Literature shows that antifungal agents incorporated into tissue conditioners are effective for the prevention and treatment of denture stomatitis [1,4]. However, drug incorporation at commercially available concentration can alter mechanical and structural properties of the material such as increased hardness, loss of the cushioning effect and distortion [4,6,7,8,9,10]. In addition, there is risk of emergence of resistant microbial strains.

Recently, use of natural compounds with inherent antimicrobial characteristics which can limit the emergence of drug resistant species has been advocated [11]. Chitosan (CS) is a biocompatible, natural biopolymer having broad spectrum antimicrobial activity [12,13]. Lee et al. explored potential use of chitosan for the treatment of denture stomatitis. They observed considerably lesser fungal colonies attached to the modified tissue conditioners and minimal effect on the viability of human gingival epithelium cells [14]. Mousavi et.al. also noted the effective inhibition of pathogenic microbes on the surface of tissue conditioner modified by chitosan nanoparticles [15]. Similarly, Saeed et al. observed fungistatic effect of chitosan-tissue conditioner formulation lasting for 7 days in a study involving comparison of a synthesized chitosan oligosaccharide (COS) with commercially available low molecular weight chitosan (CS) against *C. albicans* [16].

Although these studies show promising antifungal results, none evaluated the impact of these additives on the properties of tissue conditioners. Loss of soluble components and absorption of water has been reported for tissue conditioners when in contact with saliva and storage medium. These can result in dimensional changes, deterioration of material, increased *Candida* growth and loss of resilience of material, thus affecting the functional efficacy and clinical longevity of the material.

The present study aims to evaluate the impact of chitosan (CS) and chitosan oligosaccharide (COS) incorporation on the tissue conditioner’s hardness, sorption, and solubility in different conditioning media upon aging. It was hypothesized that the mechanical and physical properties of experimental materials would alter after aging in distilled water and artificial saliva resulting in loss of tissue healing property. The results of this study will assist clinicians to make an informed choice regarding the selection of materials for the treatment of denture stomatitis.

## 2. Materials and Methods

Synthesis of chitosan oligosaccharide (COS) was carried out by acid reflux pathway as described in literature using low molecular weight chitosan (44,886,950,000–190,000) by Sigma Aldrich, St. Louis, MO, USA, acetic acid, ethanol and acetone purchased from BDH, AnalaR, England, UK [16]. Five grams of chitosan was dissolved in 250 mL of 7% *v*/*v* acetic acid and the hydrolysis was carried out at 95 °C for 20 h by acid reflux. The hydrolysed product was dried by rotary evaporator (BÜCHI Rotavapor R-200, Allschwil, Switzerland), and de-ionized water was added. This was followed by addition of 60:40 mixture of acetone-alcohol to precipitate the chitosan Oligosaccharide. The precipitated COS was washed with acetone, filtered, and dried at 60 °C. Analysis of functional groups of synthesized oligosaccharide and commercial chitosan was performed using FTIR spectrometer (Bruker, Tensor-II, Bremen, Germany) as KBr pellet at room temperature over a wavelength range of 4000–400 cm^−1^. X-ray diffractometer (Bruker D8 Advance, Bremen, Germany) was used to record structural morphology at the scanning rate of 1°/min at diffraction angle (2θ) range of 10° to 70° at 4 kV.

Low molecular weight chitosan and synthesized COS was used to modify commercially available tissue conditioner (GC Soft-Liner TM, GC Corporation, Tokyo, Japan). Its powder is composed of polyethyl methacrylate (PEMA) and a liquid is a mixture of ethyl alcohol and an aromatic ester. Part of PEMA powder was replaced with the known effective antifungal concentrations of CS 1.02 mg (Group 1: TC-CS) and COS 0.51 mg (Group 2: TC-COS) each per gram of tissue conditioner powder [15]. The powder was milled using PQ-NQ4 Planetary Ball Mill, Pennsylvania, PA, USA, for 4 h to ensure even distribution of additives into the tissue conditioner powder [15]. GC Soft-Liner TM without drug incorporation was used as a control (Group 0: CTC).

### 2.1. Sample Preparation

Specimen were prepared by homogeneously mixing 2.2 g of tissue conditioner powder with 1.8 mL of liquid according to the manufacturer’s instructions for 30 s and pouring it into rectangular stainless-steel molds of different dimensions (100 mm × 20 mm × 10 mm for Shore A hardness test and 40 mm × 10 mm × 1 mm for sorption and solubility test). The molds were sandwiched between two glass slabs lined with acetate sheet. The whole assembly was clamped and was allowed to gel in an oven (ESCO forced convention lab oven, OFA-54-8, Singapore City, Singapore) at 37 °C for 1 h. Any flash was trimmed with Bard Parker blade # 15 after removing specimens from the mould.

### 2.2. Shore A Hardness Test

Fifteen samples (*n* = 15) per group were prepared and a baseline Shore A Hardness value was recorded. Samples were then stored at 37 °C in three different environment (*n* = 5); dry (wrapped in aluminium foil), immersed in 100 mL of distilled water (DW) and 100 mL of freshly prepared artificial saliva (AS) [17]. Shore A Hardness was performed in accordance with ASTM D 2240-05 (2010) standards [18] using Shore A durometer (Novotest TS-C, Novomoskovsk, Ukraine) at predetermined time intervals, i.e., 1, 2, 3, 5 and 7 days. At each interval, Shore A durometer was calibrated prior to each set of experiments using a set of reference blocks supplied by the manufacturer, and hardness of sample was measured on one side after a dwell time of 5 s. Care was taken to record each measurement 10 mm apart from each other and 12 mm apart from the margin of the sample. Average of recorded values at each time interval was calculated.

### 2.3. Absorption and Desorption

Thirty samples (*n* = 10 per Group) were prepared and stored in an oven (ESCO forced convention lab oven, OFA-54-8, Changi South Street 1, Singapore) until a constant weight (W1) was achieved. Then, half of the samples (*n* = 5) were immersed in distilled water and remaining in artificial saliva at 37 °C for one week. At a predetermined time, interval of 1, 3, 5 and 7 days, each sample from the respective liquid was blotted on filter paper to remove excess fluid and weighed (W2) accurate to three decimal places using a calibrated analytical balance (OHAUS, PA413, Tempcon, UK) and returned to the solution and placed in the oven.

After completing the absorption study, the specimens were desorbed by removing the samples from the respective liquid and drying at 37 ± 2 °C in the drying oven (Gallenkamp, England, UK). The samples were weighed at the same time interval as for absorption until constant weight (W3) was achieved. The percentage absorption and solubility were calculated according to Equations (1) and (2), respectively [19].

Absorption% = (W_2_ − W_3_/W_1_) × 100 (1)

Solubility% = (W_1_ − W_3_/W_1_) × 100 (2)

where W_1_ is initial weight of samples, W_2_ is weight of sample after absorption and W_3_ is final weight of sample after desorption

### 2.4. Statistical Analysis

The collected data was analysed using SPSS Version 22.0, IBM Corp. Armonk, NY, USA, One-way analysis of variance (one way ANOVA) followed by the Tukey HSD test compared the formulated tissue conditioners for Shore A hardness, percentage mean sorption and solubility. The impact of immersion media and immersion time was also analysed. The *p* value ≤ 0.05 was considered to be significant.

## 3. Results

### 3.1. Characterization

**Figure 1:**
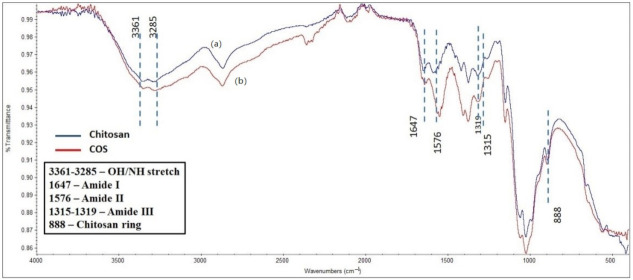
FTIR spectra of (**a**) Low molecular weight commercial Chitosan and (**b**) synthesized COS.

**Figure 2:**
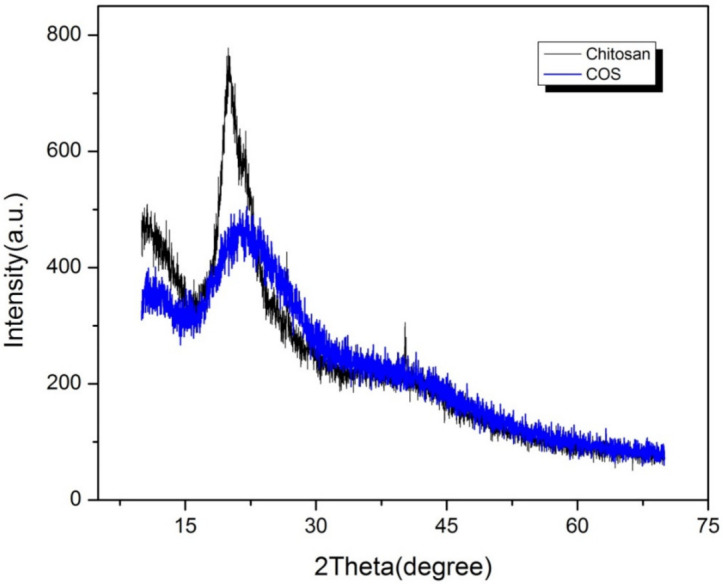
X-ray diffraction pattern of low molecular weight commercial chitosan and synthesized COS.

The IR spectrum of COS and CS were similar with no obvious peak differences as shown in Figure 1, confirming no structural changes during acid hydrolysis of chitosan. The peak at 888.07 cm^−1^ indicated the presence of a chitosan ring whereas peaks around 1647 cm^−1^, 1576 cm^−1^ and 1319 cm^−1^ represented Amide I, II and III bands respectively [16]. X-ray diffraction patterns of chitosan and COS are shown in Figure 2. Strongest reflection was noted at 20.1°. The COS had less intense and broad peak compared to CS.

### 3.2. Shore A Hardness

**Figure 3:**
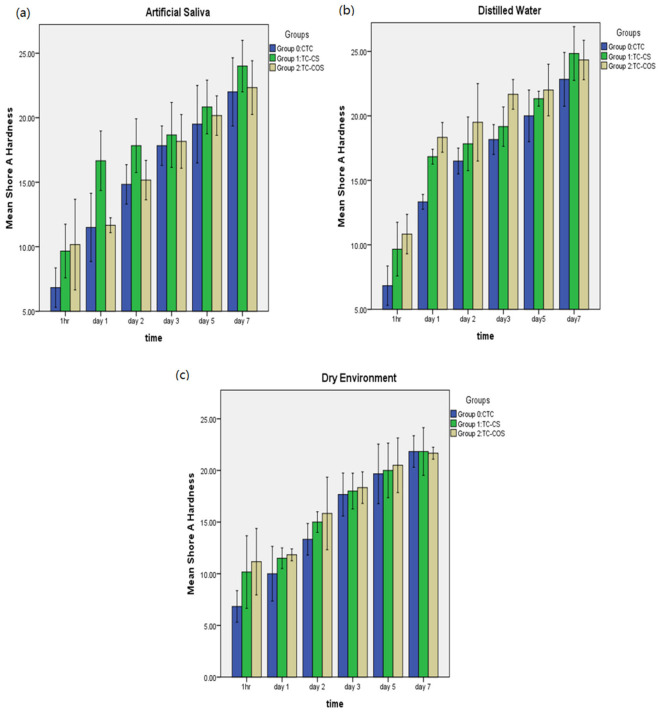
Mean Shore A hardness of tested material upon aging in various environment; (**a**): artificial saliva, (**b**): distilled water, (**c**): dry environment.

The mean Shore A hardness values for samples stored under various conditions is shown in Figure 3. For each material, durometer values were significantly increased during the first 24 h, however, upon further aging, hardness was not increased significantly. Additionally, experimental formulations had higher hardness values but there was no statistical difference between the tested materials. Samples stored in distilled water showed greatest increase in hardness followed by artificial saliva and dry condition (Figure 3).

### 3.3. Water Sorption and Solubility

**Figure 4:**
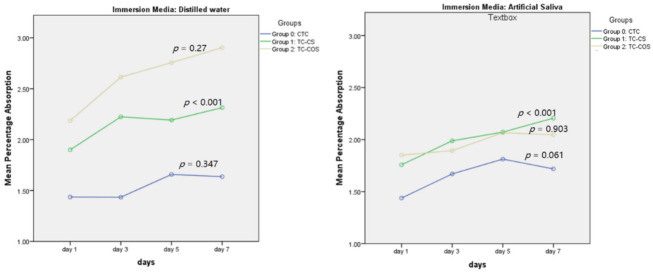
Mean percentage absorption upon aging in distilled water and artificial saliva.

**Figure 5:**
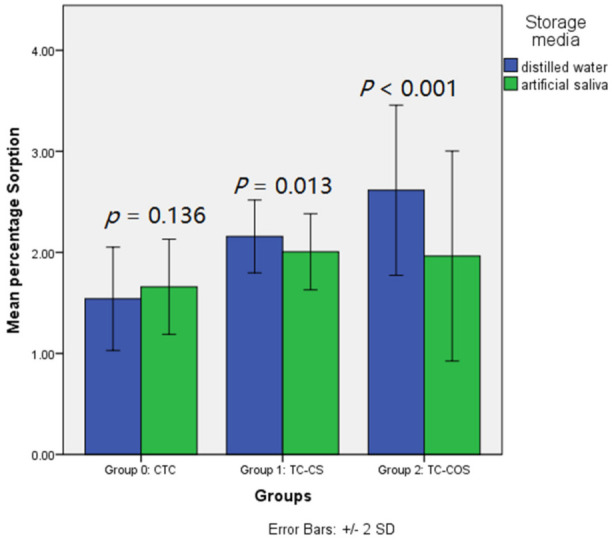
Effect of storage media on the sorption of tested material.

Net weight gain was noted for each sample upon aging when immersed in distilled water and artificial saliva (Figure 4). Significantly higher sorption values were recorded for experimental groups (Table 1). Group 0 (CTC) had statistically similar percentage sorption in each media, whereas for experimental groups, significantly higher sorption values were recorded for samples stored in distilled water compared to artificial saliva (Figure 5).

Mean solubility values of tested materials are presented in Table 2. Storage medium and aging time had significant impact on the solubility of tested materials. The solubility was observed to increase with the aging time (Table 2). In addition, significantly higher solubility was noted for materials stored in artificial saliva (Table 3). Inter-group comparison of tested materials revealed that Group 2 had highest mean solubility values in both immersion medias, followed by group 1 and group 0. In distilled water, a significant difference between Group 0 and experimental groups was noted. However, in artificial saliva, the difference was not significant between Group 0 and Group 1 as shown in Table 4.

## 4. Discussion

Tissue conditioners are used for conditioning of traumatized oral tissues [6]. To maintain functional efficacy, any dimensional changes and an increase in hardness of material are undesirable. In present study, the experimental formulations had greater durometer values compared to control group, however the changes were not significant. Findings of this research were in accordance with previous studies [20] which have demonstrated that antimicrobial agent impairs the penetration of plasticizers into the polymeric chains of the tissue conditioner and prevents formation of a soft gel. In addition, antimicrobial agents tend to increase water sorption which further increases the hardening of the material [20]. Zareshahrabadi et al. showed that the viscoelastic modulus of an experimental antimicrobial modified tissue conditioner was reduced indicating increase in hardness value [21]. In another study by Manior et al., it was concluded that terpinen-4-ol and cinnamaldehyde resulted in increased shore A hardness of experimental and control tissue conditioner over 7 days period [22]. Similarly, Urban et. al. also noted increased in hardness of autopolymerising soft denture liner (Softone) following 7 day immersion in water [20]. On contrary, Herla et al. observed reduction in hardness of acrylic-based resilient liner when modified by chitosan salts. The difference might be attributed to the variations in the salt quantity incorporated and difference in method of synthesis of chitosan derivatives [23].

Samples stored in distilled water showed greater increase in the hardness value followed by artificial saliva and dry environment. Leaching out of plasticizers in aqueous media results in hardening of material. Khaledi A et al. and Grag A. et al. observed that the leaching out of plasticizer and antifungal agent is osmotically driven [24,25,26]. Minor variation in hardness of materials immersed in artificial saliva is of greater clinical importance as this experimental set-up simulates the oral environment.

In the present study, although experimental tissue conditioners had higher shore A value over the 7 days of evaluation, the values remained clinically within the acceptable range required for conditioning of traumatized oral mucosa [27]. In addition, these values fulfilled ISO requirement for soft liners (ISO 10139-2:2009) of approximately 40 units (ASTM D2240) [18,28].

Antimicrobial agents tend to influence sorption and solubility of the material which may affect the dimensional stability and stress distribution quality of the tissue conditioners. In the present study, two storage media were compared. Distilled water was used to evaluate the diffusion processes without influence of the osmotic effects conferred by the constituents of artificial saliva [29]. Net increase in sorption value was observed for all samples immersed for a period of 7 days. Significantly (*p* = 0.018) greater sorption was noted for samples stored in distilled water. Garg and Shenoy also observed highest sorption of GC tissue conditioner in distilled water followed by sodium hypochlorite and Shellis artificial saliva [24]. Hence, previous studies supports results of current research that water uptake by material is osmotically driven [24]. Greater sorption was exhibited by experimental groups compared to the control. These results were in line with previous studies, where addition of antimicrobial agents such as nystatin and chlorhexidine in resilient materials increases water absorption [3].

Percentage solubility for all samples was higher in artificial saliva. These were in accordance with studies where solubility of plasticizers increases in ionic solutions. Highest percentage solubility was noted for Group 2 (TC-COS) followed by Group 1 (TC-CS) and Group 0 (CTC). These findings were attributed to presence of leachable antimicrobial agents in the experimental groups. Additionally, greater solubility and low molecular weight of COS was responsible for observed findings as reported previously [16]. It was also noted that solubility of chitosan was increased with reductions in pH which further enhanced leaching of CS and COS from experimental groups in artificial saliva [30].

## 5. Conclusions

Based on the results of present study, addition of chitosan and chitosan oligosaccharide in tissue conditioners at different concentrations increases hardness, sorption, and solubility of the material. However, these changes are not substantial to interfere with their tissue healing property. Consequently, our hypothesis was partially rejected. Hence the findings of present study provided important data for a potential future use of a chitosan-modified tissue conditioner for in-vivo treatment of denture stomatitis. Further studies evaluating in-vitro bond strength to the denture base materials and surface roughness of material should follow up before clinical assessment of these experimental tissue conditioners.

## Figures and Tables

**Table 1:** Comparison of tissue conditioner stored in distilled water and artificial saliva based on percentage sorption of specimens.

Immerse Media	Groups	Mean	S.D	F (*p*-Value)	Post–Hoc Tukey Test	*p*-Value
Distilled Water	Group 0: CTC (Control)	1.541	0.256	63.373 (≤ 0.001)	Group 0 vs. Group 1	≤0.001
					Group 0 vs. Group 2	≤0.001
	Group 1: TC-CS (Experimental)	2.157	0.180		Group 1 vs. Group 2	≤0.001
	Group 2: TC-COS (Experimental)	2.615	0.421			
Artificial Saliva	Group 0: CTC (Control)	1.659	0.235	5.945 (0.005)	Group 0 vs. Group 1	0.007
	Group 1: TC-CS (Experimental)	2.001	0.189		Group 0 vs. Group 2	≤0.001
	Group 2: TC-COS (Experimental)	1.964	0.519		Group 1 vs. Group 2	0.020

**Table 2:** Mean percentage solubility of specimens upon aging in different storage medium.

Groups	Duration	Distilled Water	F (*p*-Value)	Artificial Saliva	F (*p*-Value)
		Mean ± S.D		Mean ± S.D	
Group 0: CTC (Control)	1 day	0.116 ± 0.013	54.352 (*p* ≤ 0.001)	0.860 ± 0.197	13.124 (*p* ≤ 0.001)
	3 day	0.238 ± 0.024		1.092 ± 0.192	
	5 day	0.214 ± 0.063		1.092 ± 0.190	
	7 day	0.360 ± 0.035		1.424 ± 0.219	
	14 day	0.500 ± 0.065		1.678 ± 0.191	
Group 1: TC-CS (Experimental)	1 day	0.652 ± 0.255	6.106 (*p* = 0.002)	0.848 ± 0.282	6.952 (*p* ≤ 0.001)
	3 day	0.952 ± 0.274		0.960 ± 0.278	
	5 day	0.952 ± 0.284		1.328 ± 0.265	
	7 day	1.120 ± 0.235		1.416 ± 0.319	
	14 day	1.404 ± 0.181		1.656 ± 0.264	
Group 2: TC-COS (Experimental)	1 day	0.700 ± 0.209	10.909 (*p* ≤ 0.001)	1.648 ± 0.533	0.761 (*p* = 0.563)
	3 day	0.866 ± 0.275		1.790 ± 0.456	
	5 day	0.892 ± 0.265		1.728 ± 0.411	
	7 day	1.124 ± 0.361		1.874 ± 0.441	
	14 day	1.824 ± 0.356		2.112 ± 0.428	

**Table 3:** Effect of storage media on the solubility of tested material.

Groups	Immerse Media	Mean	S.D	F Value	*p*-Value
Group 0: CTC (Control)	Distilled Water	0.286	0.028	160.367	≤0.001
	Artificial Saliva	1.229	0.069		
Group 1: TC-CS (Experimental)	Distilled Water	1.016	0.068	4.654	0.036
	Artificial Saliva	1.242	0.080		
Group 2: TC-COS (Experimental)	Distilled Water	1.081	0.097	32.112	≤0.001
	Artificial Saliva	1.830	0.089		

**Table 4:** Comparison of mean percentage solubility of specimens in different storage medium.

Immerse Media	Groups	Mean	S.D	F (*p*-Value)	Post–Hoc Tukey Test	*p*-Value
Distilled Water	Group 0: CTC (Control)	0.286	0.141	39.372 (≤0.001)	Group 0 vs. Group 1	≤0.001
					Group 0 vs. Group 2	≤0.001
	Group 1: TC-CS (Experimental)	1.016	0.338		Group 1 vs. Group 2	0.790
	Group 2: TC-COS (Experimental)	1.081	0.487			
Artificial Saliva	Group 0: CTC (Control)	1.229	0.345	18.359 (≤0.001)	Group 0 vs. Group 1	0.993
	Group 1: TC-CS (Experimental)	1.242	0.399		Group 0 vs. Group 2	≤0.001
	Group 2: TC-COS (Experimental)	1.830	0.447		Group 1 vs. Group 2	≤0.001

## Data Availability

The data presented in this study is available on request from the corresponding author.
